# Entrepreneurship in care for elderly people with dementias: situated responses to NPM-based healthcare reforms in the Netherlands

**DOI:** 10.1186/s12913-023-10351-8

**Published:** 2023-12-04

**Authors:** Martijn Pieter van der Steen

**Affiliations:** Faculty of Economics and Business, University of Groningen, Groningen, The Netherlands

**Keywords:** Entrepreneurship, Alzheimer's disease, Accounting, New Public Management

## Abstract

**Background:**

Despite the great confidence of Western governments in the principles of New Public Management (NPM) and its ability to stimulate “healthcare entrepreneurship”, it is unclear how policies seeking to reform healthcare services provoke such entrepreneurship in individual institutions providing long-term healthcare. This study examines such situated responses in a Dutch nursing home for elderly people suffering from dementias such as Alzheimer’s disease.

**Methods:**

A four-year inductive longitudinal single-case study has been conducted. During this time period, the Dutch government imposed various NPM-based healthcare reforms and this study examines how local responses unfolded in the nursing home. Through interviews conducted with managers, administrators and supporting staff, as well as the examination of a large volume of government instructions and internal documents, the paper documents how these reforms resulted in several types of entrepreneurship, which were not all conducive to the healthcare innovations the government aspired to have.

**Results:**

The study records three subsequent strategies deployed at the local level: *elimination* of healthcare services; non-healthcare related *collaboration* with neighboring institutions; and *specialization* in specific healthcare niches. These strategies were brought about by specific types of entrepreneurship – two of which were oriented towards the administrative organization rather than healthcare innovations. The study discusses the implications of having multiple variations of entrepreneurship at the local level.

**Conclusion:**

Governmental policies for healthcare reforms may be more effective, if policymakers change output-based funding systems in recognition of the limited control by providers of long-term healthcare over the progression of clients' mental disease and ultimate passing.

**Supplementary Information:**

The online version contains supplementary material available at 10.1186/s12913-023-10351-8.

## Introduction

Western governments have struggled with rapidly rising healthcare costs [1], especially for long-term care for the elderly [2]. In addition, demographic changes are projected to give rise to further increases in the future [3, 4]. As a result, cost containment in healthcare has become a major issue for governments throughout the developed world [5].

An ideological shift towards the neoliberal end of the political spectrum has provided governments with new ways to take control of these costs. Such an ideological shift has been referred to as a shift from the welfare state to the “participatory society”, as it is termed in the Netherlands [6]. The former is primarily based on principles of solidarity and a strong government that organizes education, healthcare, housing, poverty relief and social insurance for all citizens as they go through different stages of life [7]. By contrast, the latter emphasizes individual responsibility, a considerable degree of choice in the market for public services, and ‘participation’ in the provision of public services, often accompanied by a withdrawal of the state as a provider of these services [6].

In the context of healthcare, a participatory society is based on the belief that individuals take responsibility for their own care needs and mobilize non-professional networks of care providers, such as family and friends, to meet those needs. The idea of a participatory society is also based on the principle that healthcare must be tailored to people’s abilities, not just their *dis*abilities. The strong reliance on individual responsibility and personal networks, and the withdrawal of the state as the primary provider of healthcare are in line with the neoliberal ideologies that Western governments have embraced.

True to these ideologies, these governments have turned to systems of “New Public Management” (NPM), which are systems of control based on the establishment of “quasi-markets” that allow contracting between independent healthcare organizations, and replace more traditional hierarchical and bureaucratic relationships as means of controlling the quantity and quality of healthcare services [8]. Drawing on management techniques from the private sector, NPM aims to stimulate a focus on operational efficiency and cost minimization, and promotes increased reliance on market-based coordination mechanisms [9]. In this sense, NPM is a set of tools and principles that are used to manage nonprofit institutions in a way that resembles commercial practice [10]. The implementation of NPM-inspired policies in the healthcare sector is particularly motivated by the expectation that they will stimulate healthcare entrepreneurship – innovative behavior in the healthcare sector that generates improvements in the quality and quantity of the healthcare process [11].

A large number of papers have examined the implementation of these policies in different sectors, including schools [12, 13], government agencies [14], and healthcare institutions, among which especially public hospitals [15, 16]. Among these and other papers, some highlight the potential that techniques informed by an NPM ideology provide a more efficient use of public resources and stimulate a more entrepreneurial mindset [17–20]. Others are more critical, suggesting that NPM introduces a managerial ideology that wrongly assumes that nonprofit organizations can be managed in ways similar to commercial organizations [21, 22].

In many countries in the Western hemisphere, NPM has informed many of the tools and instruments that governments use to control rising healthcare costs [22]. However, the use of these, often accounting-based tools as a basis for the performance evaluation of healthcare institutions has become so widespread that healthcare providers are struggling to meet all the bureaucratic demands placed on them [23]. Recent reports confirm this administrative burden [24]. As a result, there are conflicting views on the effectiveness of policies and associated accounting-based tools in stimulating entrepreneurship in healthcare, both in terms of controlling healthcare costs, and improving its quality. At one end of the spectrum, it is argued that these policies and tools, aiming to replicate commercial management principles, offer the best means of controlling rising healthcare costs through their emphasis on cost-effectiveness [19] and their ability to stimulate innovation and entrepreneurship [18]. At the other end of the spectrum, there are reports that question the efficacy of these tools, arguing that the healthcare sector is fundamentally different from the commercial sector, and therefore needs to be managed in ways that cannot be copied from other sectors [8].

One area where these conflicting views have far-reaching policy implications is in the care for elderly people with mental impairments [25]. It is estimated that the number of older people with dementias, such as Alzheimer’s disease, will almost double in the Netherlands in the next 15 years, from 290.000 to over 500.000 cases [26]. Paradoxically, this is mainly due to the increased life expectancy of people as a result of improved healthcare. As more of the population reaches the risk age for dementias such as Alzheimer’s disease, Western societies face a future with rapidly increasing numbers of elderly people with mental impairment [27]. Their responses to managing the associated costs of extra- and intramural care will largely determine their ability to provide sufficient quality and quantity of care to this vulnerable population [27]. The aim of this paper is to contribute to an understanding of how distant policies affect situated entrepreneurship, and to provide suggestions to refine these policies where necessary. I analyze the situated impact of several regulatory changes on local healthcare entrepreneurship, by drawing on an in-depth case study of a small nursing home for the elderly in the Netherlands.

To this end, the next section outlines the relevant literature on New Public Management and healthcare for the elderly. I then explain the methodological choices for this study, followed by the analysis of the case study. The paper concludes with a discussion and a conclusion.

## Literature review

### Entrepreneurship in healthcare management

Entrepreneurship is “the acts of creation, renewal, or innovation that occur within or outside an organization” [28]. The term “entrepreneurship” is used in many different ways, but it is closely related to innovation. For example, Edwards-Schachter et al. [29] argue that entrepreneurship, creativity and innovation are closely intertwined concepts. A similar point is made by Lowe and Marriott [30], who note that entrepreneurship and innovation must be considered mutual entanglements. Innovation can then be defined as “the successful development and application of knowledge and technology in the form of new technologies, products, processes, practices and services” [5]. In addition to innovation, entrepreneurship is closely associated with *creation*. Often, entrepreneurs are credited with identifying opportunities and creating something new from those opportunities [31]. This motif of creating value from previously undiscovered opportunities is the essence of many definitions of entrepreneurship such as Hitt et al.’s who refer to entrepreneurship as “the identification and exploitation of previously unexploited opportunities” [32]. The elements of innovation and the creation of value from opportunities are summarized in the definition of Ireland et al. which will be used in this paper: “entrepreneurship [i]s a context specific social process through which individuals and teams create wealth by bringing together unique packages of resources to exploit marketplace opportunities” [32].

Although entrepreneurship and innovation have mostly been associated with the private sector, governments are increasingly embracing the promise of entrepreneurship as a potential solution to the specific challenges facing the healthcare sector. Organizational renewal, innovation and the exploitation of opportunities, as outcomes of entrepreneurship, are seen as desirable attributes to combat rising costs and potentially declining healthcare service quality. There is growing recognition that entrepreneurship in healthcare organizations can spur innovation [28]. In this vein, Rowe et al. [20] speak of “corporate entrepreneurship in healthcare” to highlight how an entrepreneurial spirit can foster the identification and exploitation of opportunities in healthcare fields.

In recent decades, national governments have become more outspoken about their desire to promote entrepreneurship in healthcare management [11]. For example, in 2010, the Dutch government stated that, in order to provide affordable and accessible healthcare, the public interest would be served by placing more responsibility in the hands of private entrepreneurs [33]. However, entrepreneurship in healthcare is not only about the entry of private parties. Several papers allude to the pursuit of an entrepreneurial attitude or culture by public healthcare providers [29], with entrepreneurial managers within these organizations sometimes referred to as *intrapreneurs* [34]. There are many factors that encourage entrepreneurship in healthcare. These factors include cost pressures, government policies and regulations, increased competition, privatization, and changing societal values [34]. In fact, in recent decades, government regulatory frameworks have been put in place to encourage the provision of these factors, and thus promote entrepreneurship in the healthcare sector by entrepreneurs and intrapreneurs alike. These regulatory frameworks are underpinned by the principles of New Public Management, which are highlighted in the next subsection.

### New Public Management and accounting instruments

New Public Management (NPM) is an ideology which is characterized by “marketisation, privatization, managerialism, performance measurement and accountability” [12]. NPM has been considered notoriously difficult to define and in response, Hood [35] typifies NPM by seven ‘doctrines’, which are listed in Table 1.

**Table 1:** NPM doctrines (adapted from Hood, [35])

Unbundling of the public sector into corporatized units organized by product	More emphasis on visible hands-on top management
More contract-based competitive provision, with internal markets and term contracts	Explicit measurable standards and measures of peformance and success
Stress on private-sector styles of management practice	Greater emphasis on output controls
More stress on discipline and frugality in resource use	

In addition to being seen as a set of beliefs or ideology, NPM is also regarded a set of practices that can be observed and evaluated [8]. In many ways, the literatures about NPM as an ideology and as a set of practices do not coincide. While the former emphasizes NPM’s ambitions for efficiency and entrepreneurship [18], the latter is particularly concerned with the question of why these ambitions are often not met [10].

NPM as an ideology is based on the premise that the public sector is fundamentally incapable of delivering services in a cost-effective and efficient manner. Bureaucratic systems for controlling the allocation of public resources are considered ineffective and need to be replaced by systems which are more common in the private sector. In this sense, Osborne and Gaebler [18] advocate an “entrepreneurial spirit” in the public sector. In an attempt to evoke this entrepreneurial spirit, many countries have created institutional contexts in which a wide range of accounting tools and techniques, originating in the private sector, are prevalent. These tools and techniques include output-based funding, key performance indicators and production budget targets [36].

Academic research has been rather critical of the use of accounting measures and depersonalized metrics to bring about an NPM-based entrepreneurial attitude by public healthcare organizations. Simonet [22] found that performance contracting in the UK healthcare sector has failed to deliver on its promise of more effective use of public resources in the healthcare sector. In their analysis of NPM in Spanish hospitals, Alonso et al. [15] concluded that reforms in management models had no measurable effect on the efficiency of hospitals in Madrid. Other analyses have pointed to the negative effects of accounting-inspired reforms on healthcare entrepreneurship. For example, Newman and Lawler [21] found that such reforms have reduced the ability of healthcare managers to provide professional and clinical leadership in Australian healthcare institutions.

Almost three decades ago, Chua and Preston already questioned the use of depersonalized accounting metrics to implement healthcare reforms. They noted that “accounting-led healthcare cost control and reform initiatives around the globe is often a matter of faith. These initiatives are constituted within the rhetorics of rationality, efficiency and “free market” economics with very little evidence of their ability to achieve even these goals” [37]. Such concerns about the efficacy of accounting instruments in bringing about a more entrepreneurial, market-driven healthcare sector have been echoed in a wide variety of works [see among others [38–40]. Recently, Pflueger [41] observed that accounting interventions aimed at reforming the healthcare system fail to produce the desired results, because policymakers do not take into account that accounting is not neutral, but shapes healthcare fields as much as it describes them.

Overall, the clear mismatch between the ambitions underlying NPM-based healthcare reforms and their situated effects in individual healthcare institutions needs further investigation. National NPM-based healthcare reforms are based on the belief that the creation of quasi-markets and private sector-style accountability systems create a context that stimulates more entrepreneurial attitudes among healthcare providers. However, papers that discuss how policies informing such reforms affect individual healthcare providers tend to be mostly critical about the ways these reforms promote entrepreneurship at the local level. The mismatch between anticipated and realized outcomes is highlighted by Borkowski and Kulzick [42], among others, who claim that legislative interventions lead to more uncertainty and, consequently, fewer opportunities for entrepreneurs to innovate in the healthcare industry. This mismatch is especially significant for long-term care for elderly people suffering from dementias such as Alzheimer’s disease. Demographic projections suggest that the demand and cost of this type of care will strongly increase over the next decades [4, 27]. Therefore, there is a need for an elaborate understanding of the ways in which NPM-inspired policies stimulate specific behaviors in local healthcare providers, as these behaviors will largely determine the effectiveness of these policies in bringing down costs and promoting healthcare entrepreneurship.

Therefore, the paper seeks to answer the following research question: How do the accountability systems that underpin NPM-based healthcare reforms promote situated entrepreneurship in the care for elderly people with dementias such as Alzheimer’s disease? To address this question, the paper presents a case study in a Dutch nursing home for the elderly.

## Method

This section outlines the context and the methodological choices for addressing the aforementioned research question. The next subsection explains the reforms implemented by the Dutch government that specifically affected long-term care providers, such as the case organization. The second subsection explains the details of the case organization, a small nursing home for the elderly, followed by two subsections that outline the processes of data collection and analysis.

### Context: reforms in Dutch long-term healthcare for the elderly

Long-term care for the elderly in the Netherlands is organized through a system that categorizes individual clients according to the intensity of care that they need. In this system, potential clients of nursing homes can apply for a so-called WLZ care needs assessment by an independent government agency – the Center for Care Indications (CCI). The Center initially categorized these prospective clients into one of ten so-called “Care Intensity Packages” (CIPs). Each CIP defined a specific level of care needed by an individual client and the associated hours of care that could be provided by care organizations. Table 2 lists these 10 categories and their characteristics.

**Table 2:** CIPs and funding per 2012 (source: Zorgzwaartepakketten sector Verpleging & Verzorging 2012; PJ/11/1480/imz)

CIP	**Name**	**Characteristics**
1	Sheltered living with some supervision	Client is mostly self-sufficient, but requires some supervision because he/she cannot maintain an independent household
2	Sheltered living with supervision and care	Client cannot maintain an independent household, mostly because of somatic health issues
3	Sheltered living with supervision and intensive care	Due to extensive somatic problems, this client group needs supervision and, above all, intensive care in a sheltered living environment
4	Sheltered living with intensive supervision and comprehensive care	This client group needs intensive supervision combined with extensive care in a sheltered environment. The reasons for this can be different
5	Sheltered living with intensive dementia care	Due to serious dementia, this client group needs intensive supervision and intensive care in a protective living environment. The clients are (almost) entirely dependent on care
6	Sheltered living with intensive care and nursing	Due to serious somatic limitations, this client group needs supervision, intensive care and nursing at many times of the day in a protective living environment
7	Sheltered living with very intensive care, due to specific conditions, with an emphasis on supervision	Due to a chronic illness, this client group needs specific supervision in combination with very intensive care and nursing in a protective living environment
8	Sheltered living with very intensive care, due to specific conditions, with an emphasis on care/nursing	Due to a serious somatic disorder/disease, this client group needs specific and very intensive care and nursing in combination with supervision in a protective living environment
9	Recovery-oriented treatment with nursing and care	In addition to the condition for which the client receives (additional) treatment, the client also has other problems in terms of vulnerability and co-morbidity (such as circulatory problems, psychogeriatric disorders, musculoskeletal and/or metabolic disorders), which lead to instability, complications and reduced learning and trainability. Recovery to the level of functioning prior to the acute condition is aimed for
10	Protected stay with intensive palliative-terminal care	This client group stays in the care home for a short period of time (usually no longer than three months) in connection with imminent death, in a situation of protected stay

The financing of this form of long-term care is regulated by the Long-Term Care Act (in Dutch: Wet Langdurige Zorg or WLZ). Among other things, the Long-Term Care Act regulates individual entitlements to long-term care and their funding. As a result, recent ambitions to promote entrepreneurship in the healthcare sector have been implemented in part through changes to this Act.

In the course of 2014–2017, the first change was the elimination of CIP 1 to 3. In line with the government’s ambitions to promote the participatory society, it sought to intensify the already existing process of extramuralization. Extramuralization, as opposed to intramuralization, refers to the provision of long-term care to clients in their private homes, as opposed to a care facility. In effect, clients classified in CIP 1 to 3 were no longer entitled to reside in care facilities, but were instead required to reside in their private homes, supported by home care, if necessary. The abolition of CIP 1 to 3 is highly significant, as it underlines the Dutch government’s ambition to withdraw from lighter forms of elderly care and to leave this to informal care networks and the municipalities where clients reside.

Second, as of 2016, home care and support for the elderly in former CIP 1 to 3 categories were to be provided by their municipality. The municipalities became responsible not only for assessing clients’ care needs, but also for organizing their care. In order to organize and finance this care, the municipalities were required to operate elaborate marketplaces, where the care for each client was tendered to the lowest cost provider. For the Dutch government, the transfer of the provision of care to the municipalities made sense, because it argued that long-term care for the elderly should to be organized close to the client in order to facilitate the entrepreneurship necessary to tailor care to the client’s individual needs. However, a lack of expertise both in the assessment of clients’ needs and in the operation of care marketplaces led to supra-local cooperation between municipalities, effectively increasing the distance between the client and the organizer of care.

A third and related measure was that, in addition to abolishing CIP 1 to 3 and transferring home care to the municipalities, the government strengthened the principle of SHC – “separation of housing and care”. As early as 1995, SHC was a guiding principle for the organization of healthcare. It reflected the idea that individuals are responsible for financing and organizing their own housing as long as it can be meaningfully separated from their care needs. Arguably, such a separation could be made in CIP 1 to 3 as these clients are assumed to be in good enough health that, with appropriate support, they can be considered to be able to manage their own households. However, during the period of this case study, the SHC principle also meant that care institutions themselves had to separate care from housing. As such, they had to start acting as real estate agents for their own facilities in cases where they did not have sufficient residents classified in CIP 4 or higher.

The fourth measure was the introduction of the so-called Normative Housing Component in 2012 and beyond. This measure was another way to implement output financing for long-term care facilities for the elderly. In the past, the capital costs associated with real estate had been guaranteed by the government. In return, the government had imposed a wide variety of bureaucratic rules and regulations on the ownership of real estate by care providers. However, in an attempt to create more entrepreneurial space for care providers and to allow a greater role for market-based coordination, the Dutch government introduced the Normative Housing Component (NHC) and Normative Inventory Component (NIC). These are fixed amounts per client that are intended to cover a care provider’s real estate and inventory costs. All risks related to occupancy rates, capital financing, interest rates on capital employed and management of capital requirements and balance sheet were transferred to the individual care providers. This transfer was again motivated by the desire to create a more entrepreneurial attitude on the part of these providers. Over the years, NHC and NIC have become increasingly important metrics for funding real estate expenses, and ultimately, housing and care were fully funded through a single rate that was tied to each of the different CIP categories.

In this way, the government linked many activities, including the provision of healthcare services and real estate management activities, to a single output rate. This rate was used to fund the “production” of a long-term care facility, which was the number of clients in each CIP category multiplied by the length of their stay. This greatly simplified funding and gave greater autonomy to individual healthcare providers, which, at least in NPM ideology, encouraged healthcare entrepreneurship – innovation in the way care was delivered. Table 3 lists the resulting funding amounts associated with the production of long-term care for the elderly: the remaining CIP categories, and the components that constituted their funding.

**Table 3:** CIPs and rate components 2022 (Source: Beleidsregel prestatiebeschrijvingen en tarieven zorgzwaartepakketten en volledig pakket thuis 2022—BR/REG-22125b)

CIP	Wages	Materials	NHC	NIC	Total daily rate
4	€ 114.71	€ 40.55	€ 33.63	€ 2.74	€ 191.64
5	€ 223.18	€ 54.54	€ 33.64	€ 4.22	€ 315.59
6	€ 202.62	€ 52.74	€ 34.38	€ 4.22	€ 293.95
7	€ 279.74	€ 62.86	€ 35.44	€ 4.22	€ 382.26
8	€ 357.54	€ 75.03	€ 36.46	€ 5.40	€ 474.42
9b	€ 233.73	€ 56.56	€ 43.09	€ 5.74	€ 339.11
10	€ 405.62	€ 82.19	€ 36.46	€ 4.22	€ 528.49

### Local context of the study

This paper reports on a longitudinal case study conducted in a small nursing home for the elderly in the Netherlands. The home, anonymized as CareX, provided three main services. First, it was a home for elderly people, who did not require nursing care, but who lived there because of age restrictions. They had been admitted before the NPM-based reforms came into effect. Second, CareX was a nursing home for elderly people suffering from mental and/or somatic (i.e. physical) disorders. To this end, it included a closed ward for clients suffering from advanced stages of dementia, but most rooms were openly accessible. Finally, CareX operated a separate department to provide home care and support. This department employed home care nurses and home care specialists.

In the first year of this study (2014), CareX had a capacity of 85 residential places, 82 of which were actually filled. At that time, these clients were more-or-less evenly distributed among the different CIP categories. In 2014, CareX had a turnover of approximately €5,7 million, which came from 3 main sources of income: €5,3 million was funded by insurance companies through the Long-Term Care Act, covering the 82 intramural clients. €216.000,- were invoiced for home care services and about €200.000,- were reimbursements for property related capital expenditures, mainly interest and depreciation. In that year, CareX had an operating deficit of approximately €386.000,-. CareX operated a single property where all clients resided, and in this building were also located: a restaurant with a fully staffed kitchen, a private physiotherapy practice, a private hair salon, spaces for social activities, and offices. At the end of 2014, CareX employed 192 people, most of whom worked part-time (which is quite common in Dutch healthcare organizations), accounting for approximately 79 full-time equivalents.

### Data collection

This paper is part of a larger longitudinal study of the ways in which nursing homes for the elderly with dementia are responding to government pressure to become more entrepreneurial. This paper is based on 28 interviews conducted with a wide range of staff working in management roles in the nursing home. These employees included the director, team leaders, assistants, financial controllers and members of the supervisory board. Considering this paper’s interest, I focused primarily on managerial and administrative roles, because the Dutch government tends to initiate changes in healthcare through administrative impulses. These roles are therefore among the first to be exposed to these impulses and therefore, the respondents include all individuals in managerial and senior administrative roles in the home. The interviews are listed in Additional file 1 (attachment).

CareX was selected as a case site for the following reasons. First, it was an independent home for the elderly, rather than part of a larger conglomerate. This means that potential entrepreneurial behaviors were not impeded by restrictions imposed by other organizational units. This made these behaviors visible and available for examination at the local level. Second, the home was relatively small, which made decision-making relatively unproblematic, as internal consensus was easily reached. Third, the home was active in both intramural and extramural care. As the reforms in this paper include the transition from the former to the latter, CareX provided a context that enabled an examination of this transition for both types of care. Finally, the home’s focus on Alzheimer’s disease enables an examination of a wide variety of healthcare- and non-healthcare innovations, because this is a field typified by many innovative care options (see e.g. [43]). Because of these attributes, the case site is particularly appropriate to study situated entrepreneurship in response to healthcare reforms.

The period covered in this paper is from 2014 to 2017 inclusive, two additional interviews in 2020 and 2021, and an epilogue explaining more recent events. During the 2014–2017 period, many NPM-inspired reforms were implemented. This made it possible to observe CareX’s situated responses to these reforms. The study consisted of semi-structured interviews lasting on average 85 min. The interviews were guided by themes, which were informed by the specific focus of this paper. Additional file 2 (attachment) lists these themes. Where permission was given, the interviews were recorded and transcribed. Notes were taken during the interviews that could not be recorded, and these were refined immediately following the interview.

### Data analysis

**Fig. 1:**
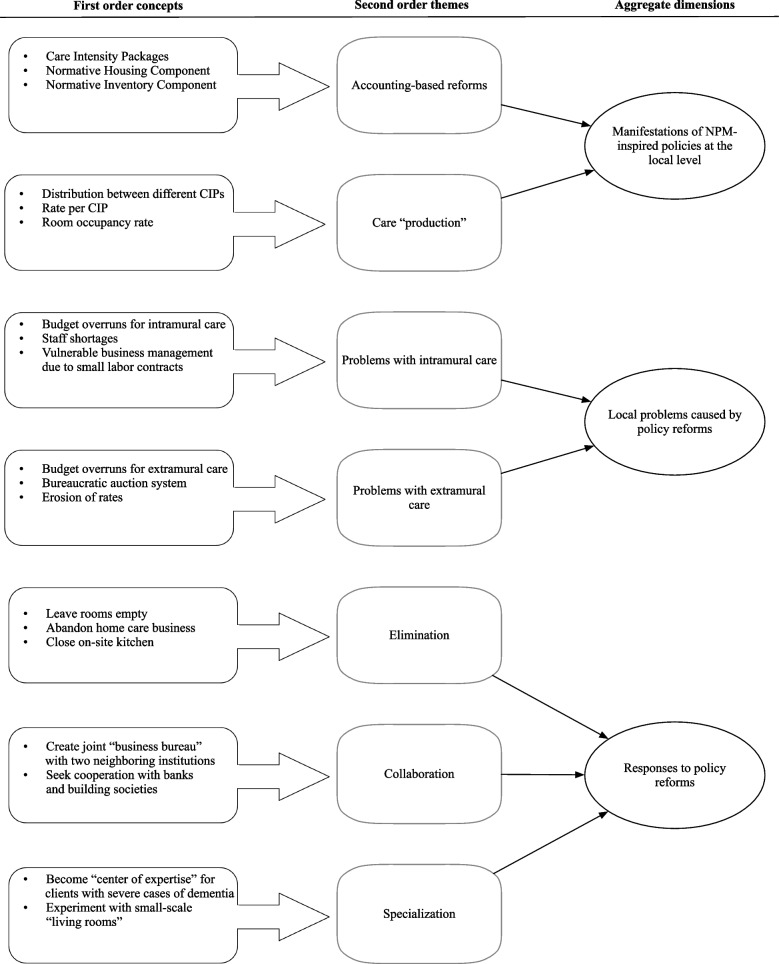
Data structure based on Gioia et al. [50]

This study is an inductive single-case study [44, 45]. Such studies are particularly appropriate for research questions aiming at building theory as they are designed to construct new theoretical concepts with greater explanatory power [46]. Following the approaches by Ruotsalainen et al. [47] and Nunes et al. [48], the case study reported on in this paper is analyzed using Gioia’s [49] three-step approach to the analysis of unstructured qualitative data [50]. Figure 1 depicts the data structure that was created in the analysis for this paper.

The figure depicts the three stages of data analysis prescribed by Gioia. In the first stage, the data were organized into first order concepts, which are empirically meaningful categories. Some examples of these first order concepts are “Normative Housing Component”, “Care Intensity Packages”, and “Leave rooms empty” (see next section). For each first order concept, Table 4 lists various illustrative quotes. In the second stage, these concepts were classified into second-order themes, which are categories that have theoretical meaning, such as “Accounting-based reforms”. Finally, in the third stage, these second order themes were aggregated into so-called aggregate dimensions, which are the building blocks of the theoretical understanding of the responses to calls for healthcare entrepreneurship. These building blocks include “Responses to policy reforms” and “Manifestations of NPM-inspired policies at the local level” and are discussed in the next section. The analysis in the next section explains the ways in which the Dutch government attempted to promote entrepreneurship in healthcare through the use of NPM-based reforms and it explains the ways in which CareX in turn responded. The resulting theoretical framework is presented in Table 5 and Fig. 3 at the end of the next section.

**Table 4:** Illustrative quotes of first order concepts

First order concept	Respondent	Date	Illustrative quote
***Care intensity Packages***	Business manager	21–01-2015	*We need to deal with incidents in our client registration system that lists the Care Intensity Packages of all our clients. And that drives a whole array of bureaucratic plans, lists and systems*
	Teamleader Care	21–01-2015	*If I have higher CIPs, I need more specialized staff. That is difficult to manage. Currently, I have this Excel sheet in which I try to keep up; how many hours do I have according to CIPs and how many do I have available from my current staff*
***Normative Housing Component***	Member Supervisory board	30–09-2014	*We will get more entrepreneurial because the NHC is no longer related to our housing costs, but rather to the number of clients we care for. That could also mean we profit from keeping our old real estate longer*
	Controller	15–04-2016	*Currently, we can use the NHC to compensate for our deficits in care. Over time, however, we have to reorganize, because NHC is barely enough to cover our new real estate*
***Normative Inventory component***	Business manager	17–06-2014	*We will bear all risks related to our inventory, as the NIC consists of norm amounts that need to cover inventory. Whether they do remains to be seen*
	Director	30–09-2015	*Over time, we will fall through the floor of our ratios. I can accept this for a limited time, but obviously, we cannot sustain shortages in funding for our inventory for a very long time*
***Distribution between different CIPs***	Director	21–01-2015	*We could reason really business-like: "how much does each CIP return" and then manage our operations accordingly. From a purely economic standpoint, I'd rather have no low CIP's*
	Project support staff member	14–10-2015	*The transition from low CIPs to higher CIPs has dislodged many things that we were working on. For example, this has great consequences for type of housing we can have and the types of staff we can employ*
***Rate per CIP***	Project support staff member	01–07-2014	*We are starting to calculate towards matching CIP's with our costs. Our business case is now based on matching our formation with the CIP's. Rates may change, and then we see that we need to act accordingly*
	Member Supervisory board	07–09-2015	*The rates have remained stable over recent years. But the government wants people to run their own households for longer. Yet, simultaneously, the rates for home care, meant for lower CIPs are dropping. So what are we to do?*
***Room occupancy rate***	Controller 2 / Interim manager	14–12-2016	*I was very surprised that in the first MT-setting where I was present, and which I could chair, that occupancy rates didn't come up: that we did not talk about how that relates to employment. The first determines our revenues, and the second determines our costs for 70%*
	Teamleader Care	21–01-2015	*Once a year, I learn how many hours my people have available to care for people. Sometimes, when a client leaves or dies, we get someone new in exactly the same CIP, so I am fine then. But when we get 5 new people in a higher CIP, that has major consequences for my care hours. So, occupancy is not just related to how many rooms are filled, but also to the kinds of CIP they are filled with*
***Budget overruns for intramural care***	Teamleader Day care	21–01-2015	*We can see this in creative activities. For people who reside in their private homes, there is a relatively large budget for creative activities and supervision thereof. The argument is of course that if you live at home, you need to take part in a lot of activities, so you don't get so expensive at later stages, so to say. But when your health deteriorates, and you end up in a home like ours, there is no real interest for policymakers to fund creative activities, except perhaps quality of life arguments, but those are not in the immediate interest of policy makers. So, you see that the system has led to a situation where the budgets for intra-mural activity supervision have been reduced a lot. I hope that we will remain somewhere in the CIP's, but it is tight*
	Business manager	17–06-2014	*We are now in the midst of the transition from intramural to extramural care. And this is immediately turned into to a cost-cutting exercise, because the municipalities that need to do this are getting less money as well*
***Staff shortages***	Teamleader Care	17–06-2014	*We are going to deliver a different kind of care, more specialized and focused on people with more severe afflictions. We need staff with a different profile for that. Currently, we may have too many people who are either educated in a wrong field or not educated enough. But we do not have enough people with the proper profile, and they are much harder to find*
	Director	21–01-2015	*It is a challenge, especially when someone is ill or on holiday. We need to replace them or ask other staff to work more*
***Vulnerable business management due to small labor contracts***	Teamleader Care	21–01-2015	*We have many very small contracts, some even 5 h per week. […] That has an impact on efficiency and quality. So where possible, we try to combine these, but that is not always possible, so it takes a lot of management*
	Controller 2 / Interim manager	14–12-2016	*Imagine, someone resigns, and I have to find an administrator for twelve hours. I will not find a capable person, who is willing to work for twelve hours. So that this too vulnerable, too risky and qualitatively, it is too unstable*
***Budget overruns for extramural care***	Member Supervisory board	30–09-2014	*We will be getting rates for home care that are severely loss-giving. We could say "these are our future clients" and justify these losses, but we cannot do this for ever*
	Project support staff member	11–11-2014	*We will experience declining rates and we need to counter the resulting budget overruns. We now have the basic rule that especially in the lower function categories, we will not extend labor contracts beyond two times, so as to avoid appointing people on permanent contracts*
***Bureaucratic auction system***	Member Supervisory board	01–06-2015	*In these auctions, all parties can tender bids at certain prices. But the consequence has been that we got really large organizations. For example, one municipality is now dealing with a large organization from another part of the country, which is very cheap because they do it very differently to us. They schedule people through computer scheduling software and they are much cheaper. They do not have a large office; they just have employees located throughout the country. And so, they can accept much lower rates*
	Business manager	21–01-2015	*The auction system really auctions clients*
***Erosion of rates***	Controller	15–04-2016	*People are getting older. And that population need more and more specialized and dedicated care. But, in contrast, there is a squeeze on our rates. So, you have to provide more care, but every time for a little bit less money. And that cannot continue, because our wages continue to rise*
	Business manager	21–01-2015	*For home care, in 2014, we received €23,97 for an hour of work. This year, the rate will be €22,11 and the expectation is that we will provide discounts on those rates as well*
***Leave rooms empty***	Project support staff member	14–10-2015	*With regards to overproduction, we need better methods and systems to predict what is coming, to get at least some insight in some of its predictable causes. Based on those types of figures, we can then decide to keep rooms empty. We have always said that we have a social remit, and we don't want to deny anyone appropriate care. So, we entered a new phase*
	Director	03–03-2014	*We calculated that we would have overproduction of about €200.000,-, last year. Fortunately, we were able to get additional funding for additional services rendered, but that is not going to happen again. So, we need to manage much stricter on occupancy of rooms, and, if needed, keep them empty*
***Abandon home care business***	Business manager	21–01-2015	*Home care is a difficult story. If we were to lose 30 intramural clients, we would need at least 60 home care clients to recoup some of the lost revenues. The rates are just that bad, because that market is highly competitive*
	Member Supervisory board	01–06-2015	*Home care is currently loss-giving. We should get rid of it, and we are thinking about it. Thus far, our thinking was that these losses may be justified, because they lead to new business later. If you are a client of our home care, and it is time to orient yourself towards more specialized intramural care, it is more likely that you think of us first. But this reasoning only goes so far as home care may become untenable, real quick, financially speaking*
***Close on-site kitchen***	Controller	15–04-2016	*The kitchen and its staff obviously are not part of "hands at the bed" workforce. So if you have to cover € 100.000,- for your kitchen, you just have to eliminate it. You need to organize differently, because it allows us to retrieve this money quite quickly, without affecting direct care. We now get ready-to-eat meals that we heat and serve*
	Director	30–09-2015	*There is a full-blown reorganization, as we need to save about ****€****450.000,-. It is going to hurt. Overhead, such as cleaning, reception, but especially the kitchen are going to suffer, because we try to keep principal care going for as long as possible*
***Create joint "business bureau" with two neighboring institutions***	Controller 2 / Interim manager	14–12-2016	*A business bureau makes a lot of sense: you are much less vulnerable, and I can get a much better sense if I deploy people in the best ways, related to their skills, salary, education and abilities. It is good to have this bureau, because up to now, there was too much discussion, too many policy changes, too many things going on. Now business management is isolated from all that*
	Controller	12–02-2016	*All those solo-functions… We are working hard to combine them and to have more people capable of doing them, just to reduce our exposure to risks. If we see that this works well, after a few months, we can start to integrate more*
***Seek cooperation with banks and building societies***	Project support staff member	21–01-2015	*We have hired a consultant who connects us to parties in the market. We have made a business case for the new real estate that we want to erect, and they will see which banks or other parties are interested and which rates they are looking for to fund our plans. […] These things are important, because the government is also withdrawing from housing, so we need parties with competencies in this area as well*
	Member Supervisory board	30–09-2014	*We need banks, but they are not eager to finance care institutions. That is a problem, but in the past, we could go to the municipality to get guarantees that would bring banks on board. Unfortunately, we do not get these anymore. The system seems to suffer from a loss in solidarity*
***Become "center of expertise" for severe cases of dementia***	Director	30–09-2015	*We want to be a regional center of expertise for Alzheimer's disease. That is our growth model. It does mean that we need to employ the right type of psychologist, behavioral experts, specialized nurses or care specialists who can work with this specific type of problem*
	Member Supervisory board	01–06-2015	*[CareX] knows a lot about dementias, because we already have many clients with Alzheimer's at different stages. We already have several "living rooms", so it is just a matter of adding a few more*
***Experiment with small-scale "living rooms"***	Teamleader Care	21–01-2015	*We just created new living rooms, with the most recent one for clients in CIP 4. I am really enthusiastic. With proper guidance and supervision, these are people who can still manage parts of a household, and that is what we do in these living rooms. There is a small team of supervisors and when they cannot manage, the care team helps out. In these rooms, people eat together, they drink coffee together. There is simply more attention for them. I have seen people really rebound and become more active and engaged*
	Director	30–09-2015	*Eight clients share a living room, and financially, that is more than sustainable, because we get to retain some return on capital. It just makes sense. We already knew that people came to us for our care, sometimes from afar. And then, real estate does not matter as much: people like living together in a good, cozy living space. People find that here and just really good care*

## Case findings

The most salient finding is that the accounting-based reforms which were intended to stimulate a more entrepreneurial attitude on the part of individual healthcare providers – an attitude that seeks to exploit previously unexplored market opportunities and create value in the form of new and improved services – produced results that were contrary to these objectives. In particular, a lack of innovation in the delivery of healthcare services was observed. This paper highlights three separate sequential processes resulting from these reforms. This section is divided into three subsections providing evidence and explanations for each of these three processes. The next subsection highlights *elimination*, which was a process of service reduction, rather than the creation of innovative new services. Then, the subsequent subsection highlights *collaboration,* the process through which CareX sought to pool administrative resources with other care providers to mitigate the negative financial impact of the prior process of elimination. The third subsection highlights a process of *specialization*, a process through which CareX’s management attempted to exploit the opportunities presented by the demography of a rapidly aging population. The fourth and final subsection, however, explains why this entrepreneurial approach by senior management was ultimately unsuccessful.

### Elimination

Table 3 shows that, in general, clients indicated with more severe stages of dementia generate higher levels of funding. This distinction is justified, because these stages of dementia require more intensive care. As explained above, the funding for CareX was based on the concept of production. However, this production was reported at a single baseline date in June. If CareX measured a lower average production during the year (for example, a higher-than-expected mortality rate resulting in a higher inflow of clients indicated in a lower CIP), it faced funding cuts in the following year. If production was higher (e.g. a higher average CIP due to a deteriorating health of a larger than expected proportion of the population), it would not be fully reimbursed for the additional costs until the baseline date of the subsequent year. CareX therefore had a strong incentive to stay as close to the agreed production as possible, but production planning was impossible when dealing with elderly people diagnosed with unpredictable diseases such as Alzheimer’s or other dementias. The director noted:

*We are a care provider, and our mission is to provide care to those in need. […] I cannot control their life or death or their rate of deterioration. Yet, the system works as if I can. (Director)*

The lack of control over life-or-death outcomes was problematic for CareX, especially since it valued moral and social considerations over the resulting financial implications:

*If a client’s condition deteriorates, he or she will be placed in a higher [CIP] and we provide more care, but this care is not always funded. […] But we have a moral and also a legal obligation to provide care. So there is an inherent mismatch between funding and care needs. (Director)*

Initially, CareX tried to renegotiate with the funding agency and, in the meantime, it attempted to close the gap between care and funding by eating into its equity. However, management understood that this would not be sustainable in the long run, because in some cases, the mismatch between funding and care was such that it threatened CareX’s existence. The organization’s equity was simply not enough to cover the lack of funding. For example, in 2014, “in a period of three months, we had the same number of deaths that usually take about a year” (Director). The timing of these deaths was critical to funding, but obviously, CareX had no control over this timing.

In general, clients entered CareX at relatively low CIP levels, and during their stay their health deteriorated until they passed away and were replaced by new residents. The impact on production, and therefore funding, measured at the June baseline, was unpredictable but profound. This impact was problematic for two reasons: (1) in the short term, CareX was unable to continuously adjust its labor force to match these fluctuations in production and funding; and (2) in the long term, CareX would need this labor force as its average CIP would rise in line with the government’s preference to admit only clients with higher CIP levels.

As part of the state’s push for more entrepreneurial healthcare management, the government did not intervene in how care production was achieved, except in cases of mismanagement. Nursing homes cannot directly influence the CIP level of new residents, but they can leave rooms empty and refuse to admit new clients when their production exceeds prior agreements. On several occasions, in attempts to manipulate the average yearly production, CareX did just that:

*This year, I had to leave several rooms for high [CIP] clients empty. This is how I try to meet prior production agreements. […] But, as a consequence, people in the region are on waiting lists longer, because we keep rooms vacant. So, there is also an ethical issue: there are directors who keep as many as 5 rooms vacant, but have waiting lists with clients to fill them. (Director)*

This was an example of *elimination* – the reduction of principal healthcare services in an attempt to cope with production-based funding that was introduced by NPM-based healthcare reforms.

Another example of elimination was the abolition of CareX’s home care department. As noted above, home care was one of the pillars of the government’s liberal policy of the participatory society – a focus on the abilities, rather than the disabilities of disabled people. As access to nursing homes had become more restricted, elderly people with minor mental or somatic disabilities were to be supported by home care workers. At the time of the case study, the responsibility for funding these services had been placed at the municipal level. With the extramuralization of CIP 1 to 3, the demand for these services increased rapidly, but paradoxically, CareX decided to abolish its home care department. It did so for a combination of reasons. First, municipalities began to operate elaborate auction systems for the home care they required for their population. A tender was issued for each individual client, and home care companies could bid to care for that client. Like all home care agencies that worked in multiple municipalities, CareX had to bid in all of the municipalities in which it operated. Not only did it have to calculate the best rates for its bids, but it also had to explain how it would meet the qualitative requirements imposed by the municipality. The overhead costs associated with these auction systems were too high to bear for an organization the size of CareX. Second, the hourly rates for home care were under severe pressure. This was largely due to the auction system, but municipalities also set maximum rates that were lower than CareX’s usual rates. These lower rates meant that CareX had to subsidize its home care with its nursing care business. This was not acceptable:

*It was a difficult decision to make, but we had the opportunity to transfer our employees to a much larger organization and we had to take it. Under these conditions, it was simply not responsible for us to continue with home care. (Director)*

CareX’s funding was based on the administrative concept of production – a concept that was not aligned with the day-to-day realities of caring for elderly with mental disease. In this context, an entrepreneurial response was cutting capacity in both nursing and home care. It is important to note that these were cuts in the principal service processes – the fee-for-service processes. Although this response lowered administrative production and thus reduced the home’s income, it mostly lowered production that would not have been funded: it brought the daily reality of caring for the elderly closer in line with optimal administrative production – the June baseline. In this way, the concept of production did not just measure healthcare outputs, it was actually constitutive of these outputs (see [41]).

As a result of the lower volume of principal services, ancillary services were also reduced, because the fixed costs of these services could no longer be absorbed by these principal services. For example, CareX eliminated its on-site kitchen, because lower occupancy rates resulted in an unfavorable allocation of the substantial fixed kitchen costs. It was replaced by external meal suppliers, whose fees were variable: each meal was billed separately. In this way, CareX sought to transform its cost structure to increase flexibility in the face of the limitations of production-based funding.

These responses were an economic necessity, because CareX could not continue to eat into its equity. They were also in line with the government’s intention to encourage a more entrepreneurial attitude at the local level. However, at this local level, this entrepreneurial attitude had mostly taken the form of eliminating healthcare services in order to counter the uncertainties surrounding output funding. Elimination was thus the situated response to the growing tensions between the unpredictable course of disease and death and the presumptions of predictability that underpinned the output funding systems.

### Collaboration

In some cases, eliminating services was not feasible. This was particularly the case for the business management of the organization. Despite the high overhead costs of maintaining a department to provide business information services, CareX could not do without, due to its own information needs and the large information requirements by the government, insurance companies and municipalities. Therefore, it established a collaboration with two neighboring care organizations. This collaboration was called “Business Bureau” and was responsible for all administrative affairs of the three collaborating organizations. These affairs included the provision of legally required information, the publication of balance sheets and income statements, and the operation of all administrative systems, with the exception of medical systems.

The Business Bureau was headed by a financial controller who explained its significance:

*For each [CIP] category, there are norms for how much care we can provide for the funding that is provided. However, [CareX] is well above these norms when you look at our total costs and revenues, and we do not yet have the information to understand in which areas we need to control our care more tightly. (Controller)*

CareX was in a difficult position. It had significantly reduced its core services, but it could not do the same for its administrative organization, because it needed more, not less information about its day-to-day operations. In this case, it opted for collaborations with other organizations to share this burden:

*What do you do when you have 24 hours of human resources capacity, 16 hours of controlling capacity, and half an hour of financial administration capacity? Business management is handled by different people who can only get part-time contracts. It makes you very vulnerable if one of those people gets sick or leaves. Combining these functions with other institutions makes a lot of sense, because, as it stands now, we are not in control and unable to deal with these administrative challenges. (Controller)*

This pooling of resources in a new shared service-center was a form of entrepreneurship, but it was limited to supporting functions – no collaborations were undertaken for the home’s principal care processes.

Another area where collaboration was sought was real estate. CareX operated a single property that dated back to the 1970’s and was at the end of its economic life. In line with the government’s preference to separate the organization of housing and care, CareX sought collaborations with several parties to provide housing to its clients. Several of these attempts involved collaborations with building societies that would construct new properties, which would be rented out to clients who would then purchase healthcare services from CareX. However, these building societies either ran into financial difficulties or withdrew from these collaborations because they were unwilling to bear the perceived risks of collaborating with a healthcare institution like CareX. As a result, CareX sought an alliance with financial institutions to finance its new property. However, since property financing was directly linked to the production of CareX through the Normative Housing Component, housing risks had been transferred from the government to CareX. This made real estate funding with commercial partners problematic:

*We have received some quotes for the new construction. However, banks seem to be reluctant to provide this financing, because it makes them dependent on production. And that is a proposition for which they want an interest rate that many homes cannot afford. (Director)*

Instead, CareX sought to extend the useful life of its real estate. Since its property was fully depreciated, NHC was more than sufficient to cover the property costs. In this way, NHC was actually subsidizing CareX’s principal processes – depreciation which was included in NHC’s standards, was not a cost to CareX, and it could invest those resources in its principal care processes instead.

However, both elimination and collaboration were short-term solutions, and management was well aware of this:

*We could not continue leaving clients’ rooms empty, eliminating core services, or organizing everything in supra-local collaborations. That was not sustainable. We had to rethink what we are about. (Director)*

The result of this strategic re-orientation was specialization.

### Specialization

The NPM-based reforms had provided CareX with two problems: (1) virtually all funding had become tied to production figures over which CareX had limited control – they were largely impacted by the course of disease and, eventually, death; (2) all business risks had been transferred to individual care institutions, which made CareX accountable for risks outside of its core competency, such as real estate and inventory related risks. In response to these *high-risk low-control* conditions, it sought for ways to reduce these risks and increase control in the long term.

**Fig. 2:**
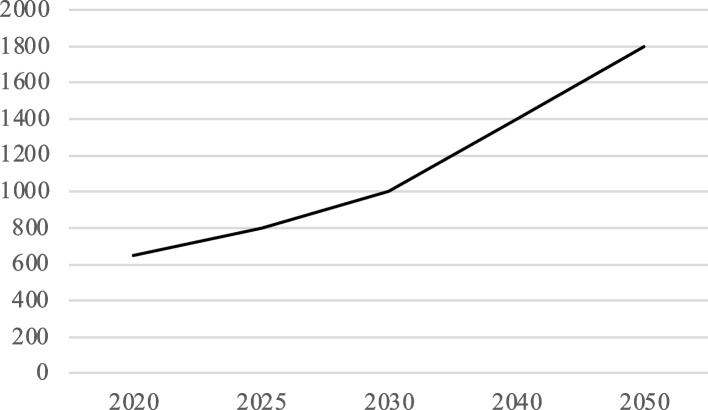
Projection of cases of dementia in CareX’s municipality

CareX used projections which showed how the number of older people with various forms of dementia will increase rapidly by 2050. For example, Fig. 2 projects a doubling of cases in the municipality where CareX was located.

CareX used these types of projections to investigate the feasibility of specializing in severe cases of dementia. The outcomes of these deliberations were summarized in a PowerPoint slide that was discussed during an internal strategy day:

*A focus on dementias has the following advantages: (1) the organization can be re-structured to provide a higher quality for this care; (2) Care processes can be managed more efficiently, because we offer fewer different types of care; (3) it is less likely that this type of care will be “extramuralized” in the future; (4) it is less likely that a mismatch between [CIP] and care delivery will occur. We expect that costs and revenues will be balanced better. (Slide pack strategy day 2017)*

Over the years, CareX had gained much experience with caring for the elderly suffering from advanced stages of dementia. It operated a closed ward and its staff was experienced in dealing with these clients. Moreover, given the government’s concern for extramuralization, a focus on clients whose housing and care could not be separated provided a shelter against future measures to further promote extramuralization. Also, in comparison to early stages of dementia, its progression at later stages is generally more unpredictable and less plannable, but because these clients are indicated in higher CIP-levels, CareX can charge higher rates to offset this uncertainty. In all, a focus on the market niche of elderly people with advanced stages of dementia would enable CareX to mitigate some of the risks it had incurred through the NPM-based reforms.

For these reasons, CareX decided to proceed with its transformation into a center of expertise for the care of advanced stage dementias. Its management exerted a lot of effort to convince local and national stakeholders, including the municipality, insurance companies and government agencies of its new strategic direction. It drew on two arguments: (1) this strategy was a prime example of healthcare entrepreneurship; and (2) all projections, such as those depicted in Fig. 2, indicated an impending shortage of capacity of care for elderly people with severe dementias. Although these stakeholders feared a rapid decline in regional capacity for the care of “lighter” cases of dementia, they reluctantly approved this strategic re-orientation.

Having a clear strategic direction was very important to CareX, because of the close relation between disease progression, property funding, and production, which all determined risk and financial results. A focus on severe cases of dementia enabled it to further develop relevant care capabilities which would also minimize the risks introduced by the NPM-based reforms. For various managers, such a focus was also desirable, because it reflected a way forward, in contrast to the service contractions that they had experienced in previous years. However, the years of these contractions had done reputational damage that was still felt:

*We lost credibility with municipalities and insurance companies, because they believed that we had become too small to survive on our own. However, given the demographics in [municipality], there was simply not enough capacity to accommodate the coming rise in dementia cases. We believed that we could expand again, once the government acknowledged this rise. […] Until that moment came, we needed to show that we were up to the task. (Director)*

In the early stages of this strategic orientation, the decline in income resulting from the decline in occupancy rates was more than offset by a slowly increasing average CIP level (in the years 2018 and 2019, CareX showed a stable yearly operating result with a decreasing capacity). So, initial results indicated that care X could survive until the demographic projections had become reality. However, CareX’s loss of legitimacy, generated by the service contractions in the earlier episodes of administrative and structural entrepreneurship, made it especially vulnerable to external shocks. Such a shock was the COVID-19 pandemic, which had a great effect on the highly vulnerable clients in institutions for long-term healthcare.

### Epilogue

In 2020, early in the COVID-19 pandemic, care institutions such as CareX were placed in lockdown. This meant that no relatives or friends could visit clients residing at the institution. In spite of this lockdown, some of CareX’s population contracted the virus and much regulatory attention was placed on the healthcare provider and its facilities. Regulators and insurance companies exerted much pressure on CareX to expedite the planned upgrades to its property. Regulators also argued that the COVID-19 crisis had revealed how vulnerable a small healthcare provider actually is as it struggled to mobilize the resources to manage the nursing home through the pandemic. These regulators, but also insurance companies indicated that they were increasingly uncomfortable with the small size of CareX. This made production negotiations more difficult:

*This was an uphill battle. Our viability became a topic in each negotiation and there was much pressure on us to explore other options. That was bitter: we had finally become innovative in our care delivery, and in the process we downsized considerably. But now, it appeared that entrepreneurship was considered conditional on size. We needed to be entrepreneurial, but only in very specific ways and according to narrow criteria. And that is really not entrepreneurship. (Member Supervisory board)*

After much internal debate, CareX reluctantly consented to a takeover by a much larger care group. Arguably, this would speed up the construction of new real estate, and bring about more synergies with the different parts of the new parent organization. Moreover, it provided CareX with more credibility with local and regional stakeholders. In 2022, the takeover had been completed. Its ambitions to become a center of expertise on its own were never met.

Table 5 summarizes the most salient findings of this analysis. It depicts the three main responses of CareX and their connection to the healthcare reforms initiated by the Dutch government.

**Table﻿ 5:** Overview of findings

Phase	Response	Type of entre-preneurship	Explanation	Relevant reform elements	Organisational areas affected
*I*	Elimination	Financial	Reduction and elimination of healthcare services to meet financial demands	Output funding: CIP’s, NHC’s and NIC’s	Principal care processes
*II*	Collaboration	Structural	Pooling of resources by creating new collaborative organizational entity	Increased emphasis on business management	Supporting management processes
*III*	Specialization	Healthcare	Repositioning in niche where existing healthcare resources can be used more effectively	Long-term demographic projections	Principal care processes

The table highlights the three types of entrepreneurship identified in CareX. The sequential order of these types of entrepreneurship was not coincidental. Rather than through bureaucratic rules prescribing the delivery of care, the healthcare reforms were introduced through the procedures of output-funding. As noted, this required CareX to align its day-to-day care efforts with the administrative concept of production on which this output-funding was based. The elimination of primary services was a form of financial entrepreneurship, which was based on its unwillingness or inability to fundamentally alter the underlying processes of care delivery:

*We could not change our operational care processes every time our funding changed. Those were well thought out and reflected our care philosophy. So, I always tried to push other buttons first, something that did not affect care for our current clients. (Director)*

This respondent’s loyalty to CareX’s existing clients and care philosophy drove him to avoid making short-run alterations to the delivery of care, other than its quantity. However, a consequence of CareX’s financial entrepreneurship was a reduction of its ability to absorb the fixed costs of ancillary services. In an attempt to save money on services that could not be eliminated, CareX engaged in structural entrepreneurship, mostly in the form of collaborations with other care institutions. However, the eventual realization that these were mostly short-term responses to fundamental, long-term changes in the healthcare domain brought about instances of healthcare entrepreneurship. This type of entrepreneurship involved the orientation of the nursing home to serve the niche of long-term care for elderly people with severe dementias.

**Fig. 3:**
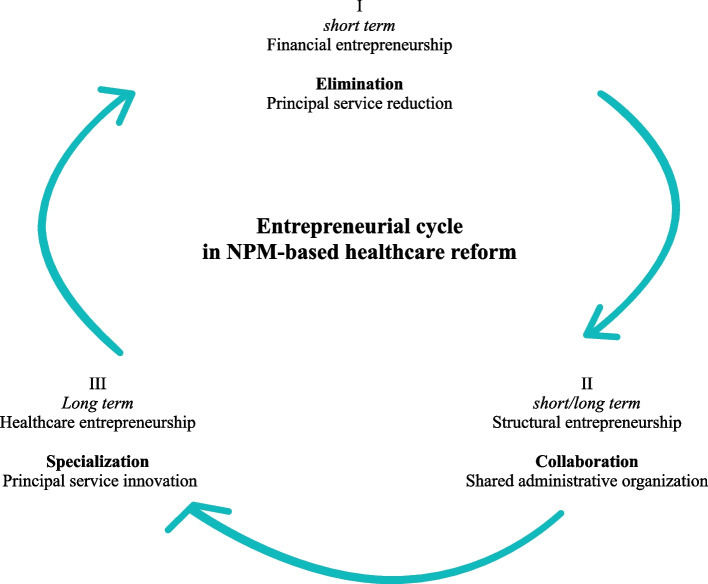
Sequential order of types of entrepreneurship

In this way, the sequential order of these types of entrepreneurship was the result of a desire to retain existing healthcare philosophies as long as possible, coupled with the fact that administrative and organizational reforms were made quicker than healthcare innovations. Figure 3 depicts this sequential order of the different types of entrepreneurship. This order can be referred to as the entrepreneurial cycle of NPM-based healthcare reform.

The significance of these findings will be discussed in the next section.

## Discussion

Figueras et al. note: *“European national policy-makers broadly agree on the core objectives that their healthcare systems should pursue. The list is strikingly straightforward: universal access for all citizens, effective care for better health outcomes, efficient use of resources, high-quality services and responsiveness to patient concerns. It is a formula that resonates across the political spectrum and which, in various, sometimes inventive configurations, has played a role in most recent European national election campaigns. Yet this clear consensus can only be observed at the abstract policy level. Once decision-makers seek to translate their objectives into the nuts and bolts of health system organization, common principles rapidly devolve into divergent, occasionally contradictory, approaches”* [51]. It is such a mismatch between policy aspirations and situated entrepreneurship that is the concern of this paper.

The paper shows how policy reforms and their associated accounting-based incentives can lead to a reduction in the quality and quantity of healthcare at the local level. Table 5 shows the different responses of CareX to these reforms. Theoretically, NPM-based policies seek to promote healthcare entrepreneurship because its characteristics are considered beneficial to the challenges of rising costs and lower service quality [22, 52]. Innovation and value creation through the exploitation of market opportunities are seen as powerful ways to address these challenges [34]. The government is attempting to encourage this entrepreneurship through the use of accounting incentives [36, 38, 41].

However, the CareX case study shows how such incentives, such as output-funding, can lead to the emergence of several types of entrepreneurship at the local level. Table 5 highlights financial entrepreneurship and structural entrepreneurship. Both types of entrepreneurship were local attempts to bring the day-to-day delivery of long-term healthcare in line with the administrative concept of production. It is important to note that neither type of entrepreneurship led to healthcare innovation, but they did constitute other forms of innovation, mostly geared towards business management and service levels. In CareX, they resulted in a lower quantity of care services and, arguably, a lower quality of ancillary services. Although a lower quantity of care services may be in line with governmental policy, the lower quality of ancillary services most likely is not and these may be unintended consequences of the chosen policies. For CareX, when these types of entrepreneurship did not provide ways to fully meet the regulatory challenges imposed by NPM-based reforms, CareX did explore ways to exploit opportunities in the long-term care market. The table lists this type as healthcare entrepreneurship, which was the ultimately unsuccessful process of transforming the delivery of long-term care. Thus, this paper suggests that the policies chosen to shape NPM-based healthcare reforms invoke multiple types of entrepreneurship, some of which may be in direct opposition to the government’s ambitions for these reforms.

Building on this suggestion, a further contribution can be made by recognizing that the order in which these types of entrepreneurship emerge may explain some of the difficulties associated with the local implementation of healthcare reforms [37, 53]. Specifically, the policies that inform these reforms work under the assumption that instruments of accountability, such as production, CIP and NHC will act as direct incentives for healthcare entrepreneurship at the local level [5, 18]. However, the results of this study show that healthcare providers will first engage in a range of alternative behaviors, because the relation between accounting stimuli and healthcare entrepreneurship is not as direct as presumed. An example of these alternative behaviors is CareX’s reduced care delivery in response to the imposition of “production” as an imperfect inscription of the day-to-day reality of this delivery [54, 55]. Although this behavior could be regarded as entrepreneurial and did improve the relation between care delivery and funding, arguably, it could be considered dysfunctional from a macro perspective, as it reduced the already strained national capacity for long-term care. However, the home’s managers viewed this response as prudent, as it protected their principal healthcare service delivery. Even when the response of reducing fee-generating services led to a need to cut costs, the managers did so by reorganizing the administrative functions, and made no attempt to intervene in the processes of healthcare delivery. In this vein, health care service delivery was highly inert.

It was only when these types of entrepreneurship proved ineffective in coping with the reforms, that CareX sought to redevelop its principal care delivery. The sequential order of these types of entrepreneurship, captured by the entrepreneurial cycle in healthcare reform in Fig. 3, was thus shaped by a strong loyalty to existing ways of delivering long-term care (see [56]). Although it is well documented that care providers can find themselves in positions of divided loyalties [57], the literature on healthcare entrepreneurship seems to overlook the fact that healthcare professionals have strong loyalties to their existing ways of care delivery and may be unwilling to abandon them for financial or administrative reasons – seeking to avoid the “moral crises” that could otherwise result [58]. The findings in this paper suggest that healthcare providers may explore opportunities for healthcare innovation only when options for alternative administrative or organizational innovations have been exhausted. Policymakers need to be aware of this inertia in healthcare reform through accounting-based regulations, as it shows how financial and administrative incentives can stimulate financial and structural entrepreneurship well before healthcare entrepreneurship may be expected.

This paper also suggests that healthcare reforms that are based on accounting-based incentives may be fundamentally flawed in one important respect. These reforms and their accompanying accounting instruments are based on the assumption that the participants in transactions have a high degree of control over their environment and the progression of clients’ disease [33]. For this reason, many of these reforms involve output funding, which places accountability for achieving these outputs, or production, in the hands of individual healthcare providers. The case study shows how the limited control over clients’ deteriorating health and the timing of their final demise created operational difficulties that were exacerbated by the systems of output-funding imposed on the organization. These accounting-based controls, such as production and CIP were imperfect inscriptions of day-to-day care delivery, because they were unable to differentiate between the controllable output of healthcare services and the uncontrollable progression of mental disease. In this way, care institutions were held accountable for the combination of healthcare service delivery *and* the unpredictable progression of clients’ disease. Consequently, CareX’s responses of elimination and collaboration targeted administrative and organizational procedures in attempts to regain control over the measurement of these uncontrollable processes, rather than the processes themselves. Arguably, these responses were innovative in many respects and may contribute to more efficient administration of healthcare service delivery, but they had limited consequences for healthcare delivery.

Finally, the case study illustrates that the specific Dutch policies of healthcare reform may be founded, at least in part, on a rhetoric of healthcare entrepreneurship, without the conditions that make such entrepreneurship possible. CareX’s decision to downsize may have been within its formal purview, but the resulting reduction of its legitimacy affected its subsequent treatment by regulators and insurance companies. Despite government rhetoric claiming the opposite, CareX’s decision-making autonomy had been limited and was eventually revoked on the basis of rather ambiguous and opaque grounds. In this sense, it may be more accurate to speak of conditional entrepreneurship as the most achievable outcome of reforms such as the one discussed here. This term emphasizes how entrepreneurship in healthcare is ultimately more restrictive than in the private sector. It also highlights the limited control that healthcare providers have over the progression of clients’ disease and eventual death. And it is this lack of control that ultimately conditions the kind of situated entrepreneurship that NPM-inspired policies can stimulate.

### Limitations

This paper reports on an inductive single case study in a Dutch healthcare organization. Such an approach is particularly suitable for theory development. However, there are some caveats that need to be considered. First, a single case study may represent an extreme case and may not be conducive to assessing the likelihood of the events observed. More studies need to be conducted to better understand the order and types of entrepreneurship in healthcare providers. Second, although this paper generalizes CareX as a healthcare provider, one may question the extent to which the understanding of the types of entrepreneurship can be extended beyond the care of elderly people diagnosed with dementias, such as Alzheimer’s disease. Alzheimer’s disease can be quite unpredictable, especially at more advanced stages. It may be that other care settings that deal with more predictable afflictions have a better match between the care that they provide and the associated output-based funding. More studies are therefore needed in care facilities that provide different forms of long-term care. Third, the recognition that healthcare providers can be entrepreneurial in different ways will have consequences for policies based on market-based ideologies. So far, NPM has been criticized for ignoring the fact that the healthcare sector is different from the private sector [22]. The findings in this paper support this observation and suggest that local healthcare providers may ultimately exhibit dysfunctional behavior as they engage in the elimination of healthcare services and collaborations that may not result in more innovative healthcare delivery. Therefore, these findings suggest the need for a better understanding of the policy implications of the complicated relationship between NPM-based healthcare reforms and the situated responses they provoke. Such an understanding may lead to more effective reforms with greater impact on local healthcare innovations and more direct benefits for patients and clients.

## Conclusion

This study contributes to knowledge about local responses to accounting-based healthcare reforms in three respects. First, it shows how these reforms bring into circulation multiple variations of situated entrepreneurship. Although entrepreneurship is mostly associated with improved healthcare quality and cost, this study identifies three types of entrepreneurship (financial, structural & healthcare) at the local level which affect healthcare quality and cost in markedly different ways. It therefore problematizes how the stimulation of entrepreneurship can address the challenges facing the care for elderly people with mental impairments. Second, the paper highlights a specific sequential order in these types of entrepreneurship, where administrators may opt to first innovate on financial metrics and organizational structures delivering ancillary services, before considering innovations in healthcare delivery itself. Although such financial and structural entrepreneurship can contribute substantially to lower overhead costs, they may potentially distract from necessary innovations in the primary process of healthcare delivery. Finally, the paper suggests that the controllability assumptions underpinning accounting-based reforms – assumptions of local healthcare organizations’ full control over the achievement of accounting targets – may be unrealistic. Especially in the context of Alzheimer’s disease, care providers have limited control over disease progression and clients’ demise, but accounting-based reforms often assume that they do. Consequently, these reforms may potentially produce unintended consequences at the local level.

### Supplementary Information

**Additional file 1.** Interviews.

**Additional file 2.** Interview themes.

## Data Availability

The qualitative datasets generated and/or analyzed during the current study are not publicly available due to a confidentiality agreement with the case organization. The quantitative data generated and/or analysed during the current study are available in the “Jaarverantwoording in de zorg” repository, https://www.jaarverantwoordingzorg.nl.
