# Supplementary material for: Entrepreneurship in care for elderly people with dementias: situated responses to NPM-based healthcare reforms in the Netherlands
Source: BMC Health Serv Res. 2023 Dec 4;23:1349. doi: 10.1186/s12913-023-10351-8 (PMC10694910; doi:10.1186/s12913-023-10351-8)
Supplement: Supplementary file 1 — Additional file 1. Interviews. [file 12913_2023_10351_MOESM1_ESM.pdf]

Appendix 1: interviews

Date	Role	duration (hrs)
03/03/2014	Director	1.5
20/03/2014	Project support staff member	0.5
17/06/2014	Teamleader Day care	1.5
17/06/2014	Teamleader Care	1.5
17/06/2014	Business manager	2.5
01/07/2014	Project support staff member	1.5
30/09/2014	Member Supervisory board	2
11/11/2014	Project support staff member	1.5
21/01/2015	Project support staff member	1
21/01/2015	Teamleader Care	1
21/01/2015	Director	1.5
21/01/2015	Teamleader Day care	1
21/01/2015	Business manager	2.5
01/06/2015	Member Supervisory board	1
07/09/2015	Member Supervisory board	0.5
30/09/2015	Director	2
30/09/2015	Policy officer	2
14/10/2015	Project support staff member	1
17/10/2015	Member Supervisory board	1
12/02/2016	Controller	1.5
14/04/2016	Member Supervisory board	1
15/04/2016	Controller	2
06/09/2016	Member Supervisory board	1
14/12/2016	Controller 2 / Interim manager	1.5
14/12/2016	Director	0.5
06/10/2017	Controller 2 / Interim manager	1.5
20/01/2020	Director	1
24/02/2021	Member Supervisory board	1
	**Average duration**	**1.41**
