# Supplementary material for: Entrepreneurship in care for elderly people with dementias: situated responses to NPM-based healthcare reforms in the Netherlands
Source: BMC Health Serv Res. 2023 Dec 4;23:1349. doi: 10.1186/s12913-023-10351-8 (PMC10694910; doi:10.1186/s12913-023-10351-8)
Supplement: Supplementary file 2 — Additional file 2. Interview themes. [file 12913_2023_10351_MOESM2_ESM.pdf]

Appendix 2: Interview themes

Interview themes

- CareX's healthcare services and delivery.
- Ways in which reforms were motivated by governmental institutions.
- Nature of recent reforms for CareX and its immediate stakeholders.
- Nature of local manifestations of reforms: accountability practices, such as performance contracting and market-based transacting.
- Experienced problems related to healthcare service delivery in relation to reforms.
- Experienced problems related to business management in relation to reforms.
- Consequences for clients' care and well-being.
- Consequences of recent reforms for extramural and intramural care.
- Local innovations in business management / other.
- Local innovations in healthcare service delivery.
- Forms and nature of entrepreneurship by managers and administrators.
- Outcomes for positioning of CareX in local healthcare markets.
